# An efficient procedure for the recovery of DNA from formalin-fixed paraffin-embedded tissue sections

**DOI:** 10.1093/biomethods/bpac014

**Published:** 2022-07-26

**Authors:** Utako Oba, Kenichi Kohashi, Yuhei Sangatsuda, Yoshinao Oda, Koh-Hei Sonoda, Shouichi Ohga, Koji Yoshimoto, Yasuhito Arai, Shinichi Yachida, Tatsuhiro Shibata, Takashi Ito, Fumihito Miura

**Affiliations:** Department of Biochemistry, Kyushu University Graduate School of Medical Sciences, 3-1-1 Maidashi, Higashi-Ku, Fukuoka 812-8582, Japan; Department of Pediatrics, Kyushu University Graduate School of Medical Sciences, 3-1-1 Maidashi, Higashi-Ku, Fukuoka 812-8582, Japan; Department of Anatomic Pathology, Kyushu University Graduate School of Medical Sciences, 3-1-1 Maidashi, Higashi-Ku, Fukuoka 812-8582, Japan; Department of Neurosurgery, Kyushu University Graduate School of Medical Sciences, 3-1-1 Maidashi, Higashi-Ku, Fukuoka 812-8582, Japan; Department of Anatomic Pathology, Kyushu University Graduate School of Medical Sciences, 3-1-1 Maidashi, Higashi-Ku, Fukuoka 812-8582, Japan; Department of Ophthalmology, Kyushu University Graduate School of Medical Sciences, 3-1-1 Maidashi, Higashi-Ku, Fukuoka 812-8582, Japan; Department of Pediatrics, Kyushu University Graduate School of Medical Sciences, 3-1-1 Maidashi, Higashi-Ku, Fukuoka 812-8582, Japan; Department of Neurosurgery, Kyushu University Graduate School of Medical Sciences, 3-1-1 Maidashi, Higashi-Ku, Fukuoka 812-8582, Japan; Division of Cancer Genomics, National Cancer Center Research Institute, 5-1-1 Tsukiji, Chuo-ku, Tokyo 104-0045, Japan; Department of Cancer Genome Informatics, Graduate School of Medicine, Osaka University, 2-2 Yamadaoka, Suita, Osaka 565-0871, Japan; Division of Genomic Medicine, National Cancer Center Research Institute, 5-1-1 Tsukiji, Chuo-ku, Tokyo 104-0045, Japan; Division of Cancer Genomics, National Cancer Center Research Institute, 5-1-1 Tsukiji, Chuo-ku, Tokyo 104-0045, Japan; Laboratory of Molecular Medicine, Human Genome Center, The Institute of Medical Science, The University of Tokyo, 4-6-1, Shirokanedai, Minato-ku, Tokyo 108-0071, Japan; Department of Biochemistry, Kyushu University Graduate School of Medical Sciences, 3-1-1 Maidashi, Higashi-Ku, Fukuoka 812-8582, Japan; Department of Biochemistry, Kyushu University Graduate School of Medical Sciences, 3-1-1 Maidashi, Higashi-Ku, Fukuoka 812-8582, Japan

**Keywords:** DNA extraction method, next-generation sequencer, clinical sequencing, formalin-fixed paraffin-embedded (FFPE) tissues

## Abstract

With the advent of new molecular diagnostic techniques, retrieving DNA from the formalin-fixed paraffin-embedded (FFPE) tissues has become an essential yet challenging step for efficient downstream processes. Owing to low quality and quantity of DNA retrieved from the FFPE sections, the process is often impractical and needs significant improvements. Here, we established an efficient method for the purification of DNA from FFPE specimens by optimizing incubation temperature, incubation time, and the concentration of a formalin scavenger tris(hydroxymethyl)aminomethane (Tris) for reverse-crosslinking. The optimized method, named “Highly concentrated Tris-mediated DNA extraction” (HiTE), yielded three times the DNA yield per tissue slice compared with a representative DNA extraction kit. Moreover, the use of HiTE-extracted DNA increased the yield of the sequencing library three times and accordingly yielded a log higher and more reproducible sequencing library compared with that obtained using the commonly used commercial kit. The sequencing library prepared from HiTE-extracted FFPE-DNA had longer inserts and produced reads that evenly covered the reference genome. Successful application of HiTE-extracted FFPE-DNA for whole-genome and targeted gene panel sequencing indicates its practical usability.

## Introduction

Formalin fixation is an essential method used in clinical diagnosis and basic biomedical research. Pathologists, for example, routinely use formalin-fixed paraffin-embedded (FFPE) tissue blocks for conclusive clinical diagnostics and research purposes. Moreover, paraffin embedding is a standard technique used in clinical and research laboratories to create FFPE tissue blocks in histological and research labs. Much of histological analysis depends on the slices sectioned from FFPE blocks. These sections can be histochemically and immunologically stained to enhance the visibility of cells of interest.

The property of the FFPE blocks to be stored for longer durations at ambient temperature makes them more cost effective than storing frozen tissues at ultra-low temperatures in terms of avoiding labor, maintenance, and storage costs. The FFPE tissue blocks are routinely archived in vast numbers and represent an extensive repository of tissue material for long-term clinical diagnostics and biomedical research. However, despite their usefulness as precious assets in biomedical research, the utilization of FFPE tissue sections as source materials for genetic and epigenetic analyses is associated with several difficulties. Many benchmarking studies compared the commercially available DNA extraction kits and customized protocols for optimal DNA extractions and identified common problems with the quality and quantity of extracted DNA from FFPE tissue sections (FFPE-DNA) [1–6]. Lower yields, progressive fragmentation, and sequence artifacts in FFPE-DNA tissue sections often times render them unsuitable for molecular genetic analysis [7–9].

Generally, DNA recovery from FFPE tissue sections can be divided into four steps; namely, deparaffinization, tissue lysis, reverse-crosslinking, and DNA purification. Of these, the former three steps are specifically performed in the preparation of FFPE-DNA. Deparaffinization is a step to remove embedding paraffin from the tissue and is usually conducted using organic solvent xylene, while a recent procedure employed a less hazardous mineral oil [10]. The mineral oil-based procedure successfully eliminated the labor-intensive steps, reduced hand-on time, and ultimately improved the yield and quality of extracted DNA [10]. Currently, many commercially available kits utilize the mineral oil-based procedure.

The tissue lysis step sometimes also serves as the reverse-crosslinking step. Optimizations of the solution compositions, pH, incubation time, and temperature have been investigated for the efficient solubilization of the fixed tissues. Many protocols employ the proteinase K treatment to digest the tissue sections efficiently. Reverse-crosslinking is conducted in parallel with or after the solubilization step. Increased temperature is believed to be preferable for the efficient recovery of DNA [11]. However, incubating DNA at high temperatures can cause DNA denaturation, degradation, and base modifications [12], resulting in lower yield and poor quality of extracted FFPE-DNA [7].

Damages in FFPE-DNA can have substantial downstream effects on sequencing analysis, mainly in detection of false-positive mutations like insertions, deletions, and single-nucleotide variants (SNVs). Therefore, to deal with the FFPE-DNA, special considerations should be made for the preprocessing of the FFPE blocks and during the library preparation itself. Removal of single-strands from the FFPE-DNA prevents detection of insertions and deletions in the sequenced reads [13]. DNA repairing with the enzymes involved in base excision repair pathway can reduce false-positive SNVs [14]. A random priming-based procedure is effective for the library preparation from fragmented short FFPE-DNA [15]. Although these methods would contribute to improved quality and yield of sequencing libraries, the better solution would undoubtedly be the preparation of the less damaged FFPE-DNA. Some studies indicated that incubation at high temperatures may also cause such damages [4, 13]. Therefore, incubation at high temperatures might not be a perfect solution for improving FFPE-DNA yields and quality. While extensive investigations have been conducted for the obtaining high-quality DNA from FFPE tissue sections, further improvements in the field are still warranted.

Similar to FFPE-DNA extraction, proteomic analysis utilizing FFPE sections has also advanced recently. The complete solubilization of proteins in the fixed tissue sections is the key step responsible for this technological advancement [16]. Protracted incubation of FFPE tissue sections at high temperatures and the coexistence of additional reagents have been previously described in literature [16–18]. Of these, the use of highly concentrated formaldehyde scavengers, such as glycine and tris(hydroxymethyl)aminomethane (Tris), to enhance the reverse-crosslinking of proteins [17] is notable. These parameters investigated for extracting proteins from FFPE tissue sections are expected to affect, independently and additively, the yield and quality of the DNA as well.

In the present study, we investigated the effects of different incubation temperatures, incubation duration, and concentrations of formalin scavenger (Tris) on the recovery of DNA from FFPE tissue sections. We optimized the conditions for preserving the integrity and maximizing the yield of DNA from FFPE tissue sections. Furthermore, DNA obtained using the optimized procedure was compared with that obtained using a commonly used extraction procedure with regard to their performance in whole-genome and targeted gene panel sequencing.

## Material and methods

### Mouse tissues, human specimens, and tissue sectioning

FFPE blocks of mouse liver were purchased from GenoStaff (Tokyo, Japan) and stored at 4°C until further use. The stored blocks were utilized in under 6 months. A fresh-frozen mouse liver was purchased from the same company and stored at −20°C until further use.

The details of the human specimens are provided in subsequent sections. We obtained ethics approval from the review board for using human specimens for DNA extraction and sequencing at Kyushu University (approval ID 822-02). Unless otherwise stated, the slices were proceeded to deparaffinization and DNA extraction immediately after the sectioning. For microscopic investigation, at least one section was prepared and stained with hematoxylin and eosin.

### Genomic DNA purification from unfixed samples

The genomic DNA extractions from fresh-frozen mouse liver, fresh-frozen human glioma tissues, and human blood were performed with DNeasy Blood and Tissue kit, QIAamp DNA mini kit, and QIAamp DNA Blood mini kit, respectively (Qiagen, Hilden, Germany).

### Comparison of FFPE-DNA extracted using different commercially available kits

**Figure 1:**
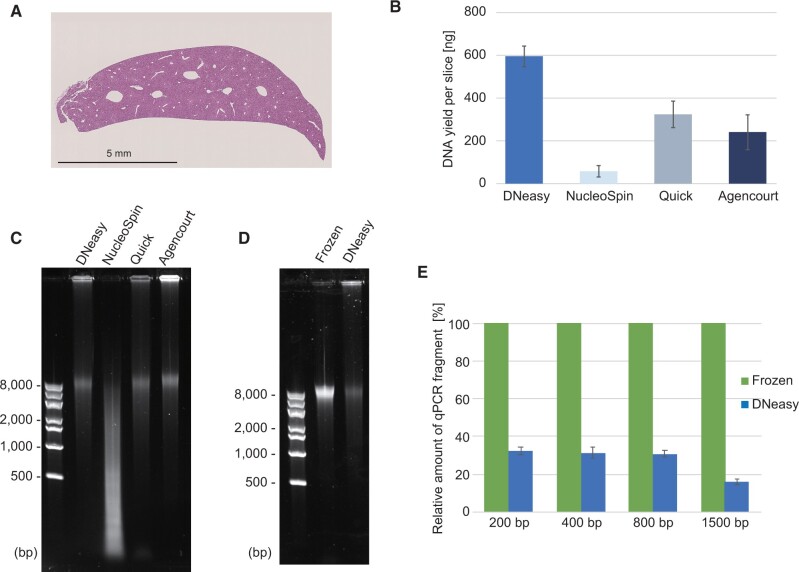
Comparison of the yield and integrity of DNA extracted with commercially available kits. (**A**) A representative image of the hematoxylin–eosin stained tissue section used for the comparison. (**B**) The mean yield of DNA extracted from FFPE sections using DNeasy Blood and Tissue kit (DNeasy) from Qiagen; Quick-DNA FFPE Miniprep kit (Quick) from Zymo Research; NucleoSpin DNA FFPE XS kit (NucleoSpin) from MACHEREY-NAGE; and Agencourt FormaPure XL DNA kit (Agencourt) from Beckman Coulter. (**C**) Image showing agarose gel electrophoresis of the extracted DNA the yields of which are presented in B. Note that the amount of DNA per well was adjusted for the visibility. (**D** and **E**) Comparison of DNA extracted from an FFPE tissue section and fresh frozen tissue. For both tissues, DNeasy Blood and Tissue kit was used. Image showing agarose gel electrophoresis of the extracted DNA (D). Comparison of qPCR amplifiable DNA fragments in the extracted DNA (E). The error bars in B and E were calculated from three independent experiments.

The FFPE-DNA extraction methods were compared using the FFPE blocks of the mouse liver. Consecutive sections of about 25 mm^2^ areas with a 10-µm thickness were prepared (Fig. 1A) and a curl was used for each DNA extraction. The purified DNA was stored at −20°C until further use.

#### Kits compared

DNeasy Blood and Tissue kit from Qiagen, Quick-DNA FFPE Miniprep from Zymo Research (Irvine, CA), NucleoSpin DNA FFPE XS kit from MACHEREY-NAGE (Hoerdt, France), and Agencourt FormaPure XL DNA from Beckman Coulter (Brea, CA) were used for extraction of FFPE-DNA. The details of the procedures used are as follows.

#### DNeasy

For the FFPE-DNA purification using the DNeasy Blood and Tissue kit, a protocol provided with the QIAamp DNA FFPE Tissue kit was used, as the two kits are supplied by the same manufacture and use common materials for DNA extractions. Briefly, a sectioned slice was immersed in 500 µL white mineral oil and incubated at 56°C for 10 min with occasional vortexing. Subsequently, the section was subjected to on centrifugation at 5000 × *g* for 2 min and supernatant containing the solubilized paraffin was discarded. This step was twice repeated. Then, 180 µL Buffer ATL and 20 µL Qiagen Proteinase K were added to the deparaffinized section, as described above and incubated at 56°C for 1 h followed by at 90°C for 1 h. The reaction was supplemented with 200 µL Buffer AL and ethanol and loaded onto a DNeasy column. The column was sequentially washed with 500 µL Buffer W1 and W2, and purified DNA was eluted using 100 µL Buffer AE. For simplicity, we call this procedure as DNeasy across this manuscript.

#### Quick-DNA FFPE Miniprep kit

A sectioned slice was deparaffinized by adding 400 μL of deparaffinization solution and incubated at 55°C for 1 min. After removing the deparaffinization solution, the section was supplemented with 45 µL each of water and 2× Digestion Buffer, and 10 µL Proteinase K, followed by incubation at 55°C for 4 h and 94°C for 20 min. After adding 5 µL of RNase A and incubating at room temperature for 5 min, 350 µL Genomic Lysis Buffer and 135 µL isopropanol were supplemented, and the reaction was loaded onto Zymo-Spin IICR Column. Then, the column was washed sequentially with 400 μL Genomic DNA Wash 1, 700 µL, and 200 µL Genomic DNA Wash 2. The purified FFPE-DNA was eluted with a 50-μL DNA Elution Buffer.

#### NucleoSpin DNA FFPE XS kit

To a sectioned slice, 400 μL Paraffin Dissolver was added and slice was incubated at 60°C for 3 min. Then, 100 μL of Buffer FL and 10 μL Proteinase K were added, and the reaction was incubated at room temperature for 3 h. Then, the reaction was supplemented with 100 μL of Decrosslink Buffer D-Link and incubated at 90°C for 30 min. After adding 200 μL of ethanol, the reaction was loaded onto the NucleoSpin DNA FFPE XS Column. The column was washed twice with 400 μL Buffer B5 and FFPE-DNA was eluted with 20 µL Buffer BE.

#### Agencourt FormaPure XL DNA

To a sectioned slice, 450 μL mineral oil was added and the slice was incubated at 80°C for 5 min. Then, 200 µL lysis solution was added and the reaction was centrifuged to collect the lysis solution and tissue at the bottom of the tube. After incubation at 80°C for 5 min, the reaction was cooled and supplemented with 30 µL proteinase K. The tissue was lysed by incubating at 55°C for 60 min and the reverse-crosslinking was performed by incubating at 80°C for 1 h. The reaction was supplemented with 5 µL RNase A and incubated at room temperature for 5 min. The DNA was captured on magnetic particles by adding 300 µL Bind solution and incubating at room temperature for 5 min. The beads were collected on a magnetic separator and washed sequentially with 400 µL Wash solution and 750 µL 80% ethanol. The FFPE-DNA was eluted with 40 µL water.

### Common deparaffinization procedure for FFPE tissue sections

Except for the FFPE-DNA preparation with commercial kits, deparaffinization of FFPE tissue sections was conducted as previously described by Lin *et al.* [10]. Briefly, sectioned slices were immersed in 500 µL white mineral oil and incubated at 56°C for 10 min with occasional vortexing. After that, the section was collected by centrifugation at 5000 × *g* for 2 min and the supernatant containing the solubilized paraffin was discarded. This procedure was repeated (usually two times) until solid paraffin completely disappeared. We did not remove the mineral oil completely to prevent evaporation of the aqueous phase in the following lysis and reverse-crosslinking steps.

### Optimization of DNA recovery from FFPE-tissue sections

#### Deparaffinization of FFPE-tissue sections

To optimize the DNA recovery procedure, we used the above-described procedure for deparaffinization of tissues.

#### The optimum temperature for reverse-crosslinking

A deparaffinized mouse liver FFPE slice was dipped in 50 µL solution containing 200 mM Tris–HCl, pH 8.0, 1% (w/v) SDS, and 5 µL proteinase K (Qiagen) and incubated at 56°C for 1 h. Reverse-crosslinking was conducted by incubating the reaction at either 70°C, 80°C, 90°C, 95°C, or 100°C. The duration periods were 1, 6, 12, 24, and 48 h for temperatures 90°C or lower, and 1, 2, 4, 8, and 16 h for temperatures 90°C or higher. After the reverse-crosslinking, DNA recovery procedure was followed as described below.

#### The optimum concentration of Tris

A deparaffinized FFPE tissue slice was dipped in 50 µL solution containing 200 mM Tris–HCl, pH 8.0, 1% (w/v) SDS, and 5 µL proteinase K, and incubated at 56°C for 1 h. Then, the reaction was supplemented with either 10, 30, 70, or 150 µL of 1 M Tris–HCl, pH 8.0; the volume was adjusted to 200 µL with 10 mM Tris–HCl, pH 8.0; and overlayed with white mineral oil. After incubation of the reaction at 80°C for 6, 12, 24, 48, or 72 h, DNA was purified using the common DNA purification protocol as described below.

#### Investigation of detergents for proteinase K treatment

A slice of deparaffinized FFPE mouse liver was dipped in 50 µL solution containing 800 mM Tris–HCl, pH 8.0, 5 µL proteinase K, and either of 1% (w/v) SDS, 1% (v/v) NP-40, 1% (v/v) Tween-20, or 1% (v/v) Triton X-100. The reactions were incubated at 56°C for 1 h, followed by at 80°C for 24 h, and the DNA was purified as the common procedure described below.

#### The optimum pH for reverse-crosslinking

Ten mouse liver FFPE slices were deparaffinized independently using the common deparaffinization procedure and collected into a tube. Then, the reaction was supplemented with 200 µL solution containing 10 mM Tris–HCl, pH 8.0, 1% (w/v) SDS, and 20 µL proteinase K. After the incubation at 56°C for 1 h, the solubilized lysate was dispensed into seven tubes by 20 µL. These tubes were supplemented with 80 µL 1 M Tris–HCl solution adjusted either at pH 4.0, 5.0, 6.0, 7.0, 8.0, 9.0, or 11.0. The reactions were overlayed with white mineral oil, incubated at 80°C for 24 h, and DNA was recovered with the common procedure described below.

### The established protocol for reverse-crosslinking of fixed tissue sections (the HiTE procedure)

The tissue slice was deparaffinized using the common procedure described above. Then deparaffinized FFPE tissue slices were dipped in 200 µL solution containing 800 mM Tris–HCl, pH 8.0, 1% (w/v) SDS, and 20 µL proteinase K. After incubation at 56°C for 1 h, the reaction was overlayed with white mineral oil and incubated at 80°C for 24 h. Then, DNA was recovered with the common procedure as described below.

### The common DNA purification procedure after reverse-crosslinking

The aqueous phase was transferred to a new tube, brought to the volume of 200 µL with water, supplemented with 4 µL 100 mg/mL RNase A (Qiagen), and incubated at room temperature for 5 min. Then, DNA was purified using the reagents and columns provided with the DNeasy Blood and Tissue kit. Briefly, the reaction was supplemented with 200 µL Buffer AL and 200 µL 100% ethanol, and the mixture was loaded onto a DNeasy column. The column was then washed with 500 µL Buffer AW1 and Buffer AW2, and purified DNA was eluted with 100 µL Buffer AE. The concentration of purified DNA was determined using the Qubit dsDNA BR kit and Qubit fluorometer (Thermo Fisher Scientific) following the manufacturer’s instructions. The purified DNA was stored at −20°C until further use.

### Quantitative polymerase chain reaction

Primers to obtain four different sizes (200, 400, 800, and 1500-bp) of amplicons and a common specific probe were designed for the mouse *Gapdh* gene (Supplementary Fig. S1). These amplicons were individually assessed for their concentration in FFPE-DNA. The primer and probe sequences used for the quantitative polymerase chain reaction (qPCR) assays are listed in Supplementary Table S2. All the primers and probe were synthesized by Eurofins Genetics (Tokyo, Japan) with OPC and QuickLC grade, respectively. Twenty microliter reaction mixtures containing 1× Luna Universal Probe qPCR Master Mix (New England Biolabs, Ipswich, MA), 0.025 units/μL of Antarctic Thermolabile UDG (New England Biolabs), 0.25 µM of each pair of gene-specific primers, 0.4 µM of probe, and an appropriate amount of DNA were prepared. The reaction was incubated at 25°C for 10 min and 95°C for 1 min. After that, 40 cycles of 3-step incubations at 95°C for 15 s, 55°C for 30 s, and 72°C for 30 s were performed for 200- and 400-bp amplicons. For 800- and 1500-bp amplicons, the 72°C incubation was extended to 90 s. The thermal cycling was performed using QuantStudio 3 (Thermo Fisher Scientific, Waltham, MA). The cycle threshold (Ct) values were converted to molar concentration, referring to Ct values calculated for known copy numbers of standards. We used genomic DNA extracted from non-fixed tissue as the standards of known copy numbers. The qPCR measured amplicon concentration was normalized with the input FFPE-DNA amount measured by the Qubit dsDNA BR kit. The DNA integrity index was calculated by dividing the molar amount of the 800-bp fragment by that of the 200-bp fragment.

### General procedure for sequencing library preparation and Illumina sequencing

If required, DNA fragments (100–500 bp) were prepared using Focused-ultrasonicator S220 (Covaris, Woburn, MA) with microTUBE AFA Fiber Pre-Slit Snap-Cap. The sequencing library was prepared using the ThruPLEX DNA-Seq Kit (Takara Bio Inc., Kusatsu Japan). The library yield was measured using the Library quantitation kit (Takara Bio Inc.). Small-scale sequencing was performed using the Illumina MiSeq with MiSeq Reagent Kit v3 (150 cycles) in the paired-end mode of 2 × 75 cycles. Large-scale sequencing was performed using the HiSeq X Ten by Macrogen Japan Corp. (Tokyo, Japan) in the paired-end mode of 2 × 150 cycles.

### FFPE-DNA preparation from normal human lymph node and sequencing library preparation

FFPE-DNA extractions from tissue blocks kept for varying storage periods were conducted using human normal lymph nodes collected for the lymphadenectomy from the colorectal cancer patients. Two consecutive tissue slices were used for the tissue lysis and reverse-crosslinking using the DNeasy or the HiTE protocols. Three different FFPE blocks were prepared for each storage period. The mean and error yields of FFPE-DNA were calculated from the yield of three blocks from the same storage period. For each FFPE-DNA, sequencing library was prepared using 2 ng DNA without additional fragmentation. Additional library was prepared using FFPE blocks of short storage periods (1 month, 6 months, and 1 year) after FFPE-DNA fragmentation with focused-ultrasonicator S220 as described above.

### Preparation of fixed budding yeast nuclei

S288C cells (Biological Resource Center at the National Institute of Technology and Evaluation, NBRC1136) were inoculated into 50 mL YPD medium (1% yeast extract, 2% Bacto peptone, and 2% dextrose) and cultivated with vigorous shaking at 30°C overnight. The cell suspension was sub-cultured in 1 L of YPD at 30°C for 4 h with shaking. Cells were collected via centrifugation at 2,000 × *g* for 15 min, washed twice with 50 mL water, and resuspended in 20 mL 1 M sorbitol. To the cell suspension, 20 µL β-mercaptoethanol and 20 mg Zymolyase 100T (Seikagaku Kogyo Co., Tokyo, Japan) were added and incubated at room temperature for 15 min to degrade the cell wall. Then, the spheroplasts were washed twice with 20 mL 1 M sorbitol and resuspended in 20 mL 1 M sorbitol. Fixation was performed by adding 240 µL of 38% formaldehyde (Wako Chemicals, Osaka, Japan) to the resuspended spheroplasts and incubating at room temperature for 5 min. The fixed spheroplasts were washed twice with 20 mL 1 M sorbitol and were pelleted via centrifugation at 5,000 × *g* for 5 min. The spheroplasts were then resuspended in 10 mL of nuclei preparation solution (10 mM HEPES-KOH, pH 7.5, 10 mM NaCl, 3 mM MgCl_2_, and 0.5% [v/v] Nonidet P 40 substitute) and incubated at room temperature for 5 min. The nuclei were collected via centrifugation at 5,000 × *g* for 5 min and washed twice with 10 mL nuclei wash solution (10 mM HEPES-KOH, pH 7.5, 10 mM NaCl, and 3 mM MgCl_2_). The nuclei were resuspended in 10 mL nuclei wash solution, of which 500 µL was dispensed into 20 tubes and stored at −80°C until further use.

### DNA recovery from fixed budding yeast nuclei and whole-genome sequencing

A frozen nuclei suspension was thawed, divided into two tubes, and pelleted via centrifugation. Then these two tubes were used for DNA extraction with DNeasy and HiTE, respectively, as described above. The purified DNAs were fragmented with sonication and sequencing libraries were prepared from 50 ng DNA. The sequencing was performed using the MiSeq system.

### Whole-genome sequencing of a retinoblastoma case

A section of 10 µm thickness was prepared and adhered to a glass slide with baking. Multiple slides were prepared using consecutive slices of an FFPE block. These slides were subjected to deparaffinization and rehydration with conventional procedures. Then, a slide was randomly chosen for hematoxylin-eosin staining, and corresponding to the stained slide, the tumor areas were marked on the unstained slides by a pathologist. The tumor area specified was scratched with a spatula and transferred into a test tube. One slide preparation was used for each FFPE-DNA extraction. After the scratching, the slide was stained with hematoxylin-eosin and visually confirmed for the scratched area. Then, FFPE-DNA was extracted using the DNeasy or the HiTE procedures. Sequencing library was prepared using 50 ng DNA and sequencing was performed using the HiSeq X Ten system.

### Target panel sequencing of glioblastoma cases

Consecutive sections of 10 µm thickness were prepared and FFPE-DNA was extracted using DNeasy or the HiTE protocols, wherein a section was assigned for each preparation. Then, sequencing library was prepared using the DNA amounts indicated in Table 1. The xGen Pan-Cancer Hybridization Panel from Integrated DNA Technologies (Coralville, IA) was used for the target panel enrichment, followed by small-scale sequencing with the MiSeq system.

**Table 1:** Cancer gene panel sequencing of two glioblastoma cases

Case	Mutation type	Storage period (years)	Sample type	DNA extraction method	DNA yield per slice (µg)^a^	First experiment			Second experiment		
						Input DNA (ng)	Library yield (fmol)	Mean coverage^b^	Input DNA (ng)	Library yield (fmol)^c^	Mean coverage^b^
1	*BRAF* V600E	4.4	Blood^d^	QIAamp	–	30	279.8	86.3		–	–
			Fresh frozen	QIAamp	–	30	84.1	55.6		–	–
			FFPE	HiTE	3.25	30	9.9	19.1	1600	67.7	119
			FFPE	DNeasy	0.97	30	4.2	6.2	980	17.4^e^	29.3
2	*IDH1* R132H	5.7	Blood^d^	QIAamp	–	30	202.8	91.1		–	–
			Fresh frozen	QIAamp	–	30	205.4	83.5		–	–
			FFPE	HiTE	0.96	30	18.7	14.1	980	77.4	77.4
			FFPE	DNeasy	0.30	30	9.1	5.5	130	10.8^e^	20.5

a DNA yield was determined with the Qubit dsDNA BR kit. The mean value for the two experiments is shown.

b Mean coverage of target regions (after removal of duplicated reads).

c Yield of library prepared in an attempt to acquire the same amount of library with the fresh frozen tissue sample.

d DNA obtained from peripheral blood of the same patient (no mutation control).

e Because of limited DNA yield, a comparable amount of library as obtained from fresh frozen samples could not be prepared.

### Agarose gel electrophoresis

Purified DNA was analyzed using conventional agarose gel electrophoresis or the E-Gel Ex system (Thermo Fisher Scientific). SYBR Gold-based signal detection was employed for both analytical systems and gel images were taken using the ChemiDoc system from Bio-rad (Hercules, CA).

### Analysis of sequenced reads

The sequence reads were preprocessed and mapped on the human reference genome, hg38, using the CLC Genomics Workbench (Qiagen). The same platform was used for the following analyses.

The alignments were exported from the CLC Genomics Workbench in SAM format and converted to BAM format utilizing samtools [19]. The genomic coverage was calculated using genomeCoverageBed of bedTools [20] and the exported bedGraph file was converted to bigWig format using bedGraphToBigWigand of Kent Utilities [21]. Genome-scale data were visualized using the Integrative Genomics Viewer (IGV) [22]. The statistics of coverage were calculated using an in-house software. Data visualization was done using Microsoft Excel and ggplot2 [23].

## Results

### Comparison of commercial kits for DNA recovery from FFPE tissue sections

We first compared four commercially available kits to assess the quality of DNA recovery from FFPE tissue sections. DNeasy, Quick-DNA FFPE Miniprep, NucleoSpin DNA FFPE XS kit, and Agencourt FormaPure XL DNA were selected because these kits are well reported in literature or are easily available. Using an FFPE block of the mouse liver as a model specimen (Fig. 1A), FFPE-DNAs were extracted. The DNA yields varied depending on the kit used (Fig. 1B) and the size distribution of DNA fragments was also different (Fig. 1C). DNeasy outperformed the other kits with respect to the yield of FFPE-DNA obtained in our hands (Fig. 1B). While NucleoSpin produced highly degraded DNA, the other three showed similar size distributions among the extracted DNA fragments (Fig. 1C). As generally acknowledged, the quality of DNA extracted from FFPE tissue sections (FFPE-DNA) was poorer than of the DNA prepared from fresh frozen tissues (FF-DNA): wherein all FF-DNA loaded went into the agarose gel, a sizable amount of FFPE-DNA stayed in the well (Fig. 1D), which indicated incomplete reverse-crosslinking of DNA.

Next, we compared the amounts of PCR-amplifiable DNA fragments in the FFPE-DNA using qPCR to determine the proportions of useable DNA for molecular genetic analysis. For this assay, primer pairs amplifying 200, 400, 800, and 1500 bp fragments were designed to compare the DNA integrity (Supplementary Fig. S1A and Supplementary Table S1). As shown in Fig. 1E, the recoveries of PCR-amplifiable fragments from FFPE tissue sections were in the range of 15.9–32.5% of those recovered from the non-fixed ones, emphasizing the need for a more efficient procedure for recovery of DNA from FFPE tissue sections.

### Incubation of FFPE tissue sections in concentrated Tris buffer improves the recovery and integrity of DNA

Complete tissue solubilization is vital for efficient FFPE-DNA extraction, wherein using harsher conditions can cause tremendous deleterious effects on downstream DNA analysis. To find optimal conditions for DNA recovery from FFPE tissue sections, we started optimizing the duration and temperature of reverse-crosslinking of FFPE tissue sections prior to DNA extraction. Due to the use of proprietary solutions in the commercially available kits, we avoided using them and performed our study using reverse-crosslinking in 200 mM Tris buffer adjusted at pH 8.0 at room temperature with hydrochloric acid.

**Figure 2:**
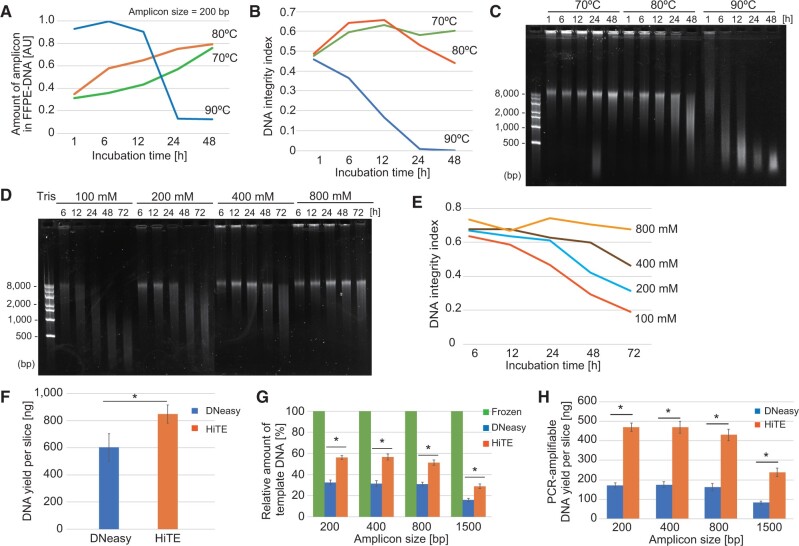
Optimization of the conditions for DNA extraction from FFPE tissue sections. (**A**–**C**) Effect of temperature and duration of incubation on reverse-crosslinking of FFPE tissue sections. The qPCR-based quantitation of 200 bp fragment (A), DNA integrity index (B), and the agarose gel image (C) are shown. (**D** and **E**) The improved DNA integrity with increasing concentration of Tris agarose gel image of extracted DNA (D) and DNA integrity index (E) are shown. (**F–H**) Comparison of DNA yield (F), qPCR-based quantitation of various amplicons (G), and DNA yield per slice (H) for DNA extracted using HiTE and DNeasy. Asterisks indicate significant (*P* < 0.05) differences between the indicated datasets (F, G and H, paired *t*-test). The error bars in F–H were calculated from three independent experiments.

While the reverse-crosslinking proceeded at 90°C or higher temperatures, severe loss of DNA integrity was observed with increased incubation time; wherein with the longer incubation, higher DNA fragmentation was obtained (Supplementary Fig. S1B). Likewise, the DNA integrity index decreased after incubation at high temperatures (Supplementary Fig. S1C). These results indicate that DNA degradation predominates reverse-crosslinking at 90°C or higher temperatures, whereas temperatures at 80°C or less would effectively maintain the DNA yield and integrity (Fig. 2A and B). However, at lower temperatures of 70°C or less, reverse-crosslinking was inefficient: stacking of the recovered DNA in wells of agarose gel was observed even when the FFPE sections were incubated for 48 h (Fig. 2C and unpublished data). Based on these results, we chose 80°C for further optimizations.

Since formaldehyde scavengers reduce the amount of free reactive formaldehyde to accelerate reverse-crosslinking [17] and prevent harmful side reactions, we next investigated the effects of Tris concentration on the preservation and reverse-crosslinking of DNA. As shown in Fig. 2D, the integrity of DNA was improved with the increasing concentration of Tris used at the reverse-crosslinking step. Furthermore, the DNA integrity index did not decrease over time at higher than 800 mM Tris concentrations (Fig. 2E), moreover, almost no loss in DNA integrity was observed even under incubation extended for 72 h. The optimal of pH 8.0 was observed for DNA integrity and recovery (Supplementary Fig. S1D and E). While the coexistence of Nonidet P-40 in the proteinase K treatment step caused a slight loss in DNA yield and integrity, SDS, Tween 20, and Triton X-100 did not affect the DNA recovery (Supplementary Fig. S1F and G).

Based on these observations, we identified an effective reverse-crosslinking condition for DNA extraction from FFPE tissue sections (see “Material and Methods” section) and named it HiTE as an abbreviation for highly concentrated tris-mediated DNA extraction. HiTE outperformed DNeasy in both mass DNA yield per slice (40.8% increase in yield, Fig. 2F) and amplifiable DNA amount in unit DNA (39.9–45.0% increase dependent on the size of amplicons, Fig. 2G). Collectively, the recovery of amplifiable DNA from a single FFPE tissue slice employing HiTE improved to 230–260% of the that obtained with DNeasy (Fig. 2H).

### Improved library yields from FFPE sections with HiTE

**Figure 3:**
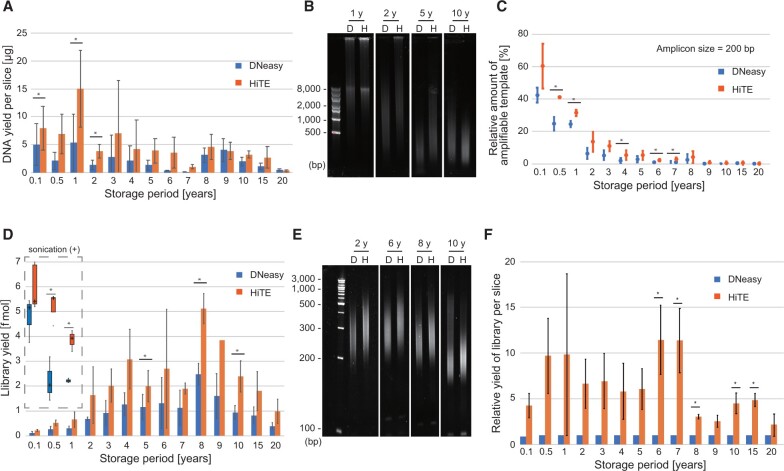
Comparison of the yield and integrity of DNA extracted from human FFPE tissue sections subjected to storage for different periods. (**A**–**C**) The DNA yield per slice (A), representative images of DNA electrophoresed on agarose gels (B), and amount of 200 bp fragment in the DNA measured using a qPCR-based assay (C) compared vis-à-vis the storage period. (**D** and **E**) The yield of sequencing library (D) and fragment size of amplified libraries (E) compared for DNA extracted using DNeasy and HiTE. For FFPE-DNAs extracted from blocks stored for a period of 1 year or less, the library yields with sonicated DNA are also shown (marked with dashed line). (**F**) The calculated yield of sequence library per FFPE tissue section compared for DNA extracted using DNeasy and HiTE. Asterisks indicate significant (*P* < 0.05) differences between the indicated datasets (A, C, D and F, paired *t*-test). The error bars in A, C, D, and F were calculated from three independent experiments.

Next, we compared the efficiency of HiTE and DNeasy by determining the yield and integrity of DNA extracted from FFPE tissue sections of clinical samples. For this comparison, we used human normal lymph nodes collected for the lymphadenectomy from the colorectal cancer patients (Supplementary Table S2). The storage periods of the FFPE tissue blocks varied between 1 month to 20 years (Supplementary Table S2) and all the blocks were stored at room temperature until the tissue sectioning. As expected, the quality of the tissue sections varied among different samples (Supplementary Fig. S2A). The yields of FFPE-DNA showed a decremental trend depending on the length of the storage period (Fig. 3A). The FFPE-DNA yield was generally higher with HiTE than with DNeasy, wherein 1.3–20.1 times more DNA was recovered with HiTE than DNeasy (Fig. 3A). In addition, the fragment size of the purified DNA obtained was larger with HiTE than with DNeasy in most cases (Fig. 3B), suggesting that DNA degradation occurs not only during the storage but also during the DNA purification procedures. We also compared the DNA integrity using the qPCR-based assay to assess a similar effect (Fig. 3C). These results supported that HiTE is superior to the frequently used DNA extraction kit for the FFPE-DNA yield.

We next prepared sequencing libraries from the FFPE-DNA. A conventional ligation-based method was used for this comparison. We employed only two cycles of amplification, a minimum number of PCR amplification cycles to complete the library structure, and to compare the net yield of adaptor tagging of the DNA recovered from FFPE tissue. The yields of the sequencing library were good for FFPE sections stored for 2–10 years, with the yields showing gradual decrease with storage periods of longer than 10 years (Fig. 3D). Alternatively, the size distributions of the obtained libraries were shortened using the FFPE-DNA extracted from extended storage periods blocks (Fig. 3E) and low library yields were observed for FFPE-DNA extracted from tissues with short storage periods of less than 1 year (Fig. 3D). This can be due to the larger fragments of FFPE-DNA extracted from short storage blocks than the optimal size used for the library preparation kit (Fig. 3B). Indeed, when fragmentation was performed, the library yields were drastically improved for these FFPE-DNA (Fig. 3D).

The library yields from the same amount of starting DNA were generally higher with HiTE than with the DNeasy for all the storage periods, wherein 1.7–2.6-times higher yield was observed using HiTE compared with that obtained from DNeasy (Fig. 3D). In addition, the fragment size of the qPCR-amplified libraries was larger with HiTE than DNeasy (Fig. 3E). As the DNA recovery and library yield were better with HiTE than with DNeasy, the relative yield of a library per tissue section was calculated to be nearly one log higher with HiTE than with DNeasy (Fig. 3F). The libraries were sequenced in a paired-end mode (150 × 2 cycles; ∼1× genomic coverage per sample). Although mapping rate and insert size gradually decreased with the increased FFPE storage period (Supplementary Fig. S2B and C), HiTE always outperformed DNeasy in these parameters (Supplementary Fig. S2D and E). Together, these results indicate higher effectiveness and efficiency of HiTE procedure for library paration from FFPE-DNA.

### Improved coverage uniformity of FFPE-DNA extracted with HiTE

To understand the differences in the DNA yields between HiTE and DNeasy, we compared the sequenced reads of the FFPE-DNA libraries prepared using the two methods. Because sufficiently rich coverage would be required for a detailed comparison of mapped reads, we chose budding yeast cells as a model system to obtain deeper reads at a reasonable cost.

**Figure 4:**
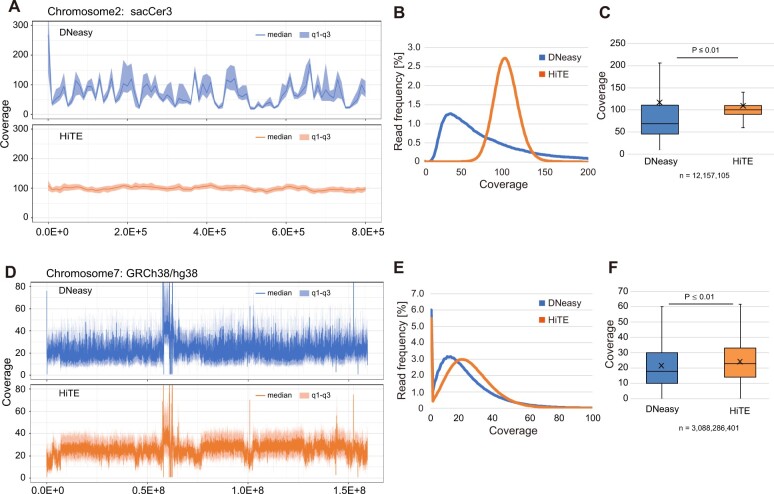
HiTE produces reads that are more evenly mappable to the reference genome. (**A–C**) DNA recovered from fixed budding yeast nuclei with DNeasy and HiTE are compared. (**D–F**) Comparison of DNA extraction from scratched tumor cells in a retinoblastoma tissue section (Supplementary Fig. S3) using DNeasy and HiTE. The representative genome browser shot (A and D), the distribution (B and E), and box plot (C and F) of mapped read coverage are shown. *P*-values were calculated using Welch’s *t*-test (C and F).

We observed one log difference in FFPE-DNA yield between the methods, wherein the DNA yield was determined as 8.7 µg using HiTE and 0.77 µg using DNeasy (Supplementary Fig. S3A). The library yield with HiTE (192 fmol) was also more than twice the yield of that obtained with DNeasy (96 fmol), starting with the same amount of FFPE-DNA (50 ng) (Supplementary Fig. S3B). The differences between DNeasy and HiTE were also observed in the quality of sequenced reads. We could not observe any difference between HiTE and DNeasy in the insert size (Supplementary Fig. S3C). However, there was a big difference in the uniformity of the read coverage. As shown in Fig. 4A, while a strongly biased coverage was observed with DNeasy, the read depth was flat across the chromosome with HiTE, even when the read numbers between the two DNA extraction methods were adjusted. The distributions (Fig. 4B) and box plots (Fig. 4C) of coverages also represented an evenness of the mapped reads with HiTE.

The difference in the read coverage observed between the two procedures using the budding yeast model showed impressive outcomes. We speculate that if a similar pattern is obtained for human tissue sections, HiTE would be a promising tool for molecular genetic analysis of FFPE sections. Therefore, we conducted the same analysis using a human tissue section. A slice was prepared from an FFPE block of retinoblastoma tissue (storage period: 1 year) and stained with hematoxylin and eosin. The scratched tumor areas were used for DNA extraction with HiTE and DNeasy-based FFPE-DNA purification procedures, and sequencing libraries were prepared without sonication. Similar results were obtained for the human tissue sections as those obtained from the budding yeast model. The DNA yield with HiTE (3.0 µg) was five times larger than with DNeasy (0.6 µg) (Supplementary Fig. S3H) and the library yield with HiTE (12.9 fmol) was approximately three times the yield obtained with DNeasy (4.4 fmol), wherein the library preparation was started using equal amounts of DNA (50 ng) (Supplementary Fig. S3I). Accordingly, a nearly one-log higher library yield per slide was obtained with HiTE than that obtained with DNeasy. In this case, the insert size was longer with HiTE than with DNeasy (Supplementary Fig. S3J). The effect of the difference between the two DNA preparation methods on DNA sequencing was very clear: we could see the same trends of mapped read coverage between HiTE and DNeasy for FFPE tissue sections (Fig. 4D–F). Although the local read coverage was more even with HiTE than with DNeasy, HiTE showed some megabase-sized low read coverage regions (Fig. 4A and D and Supplementary Fig. S4), indicating that some genomic domains are resistant to the lysis conditions used in the current version of HiTE, thereby highlighting the scope for improvements. The regions with relatively lower read coverage were found to correlate with the signals of heterochromatin markers (Supplementary Fig. S4) [24]. These results indicate that DNA extraction from FFPE tissue sections is more uniform and efficient with HiTE than with the frequently used DNA extraction method.

### Cancer gene panel sequencing of glioblastoma tissues with known mutations

Recently, targeted gene panel sequencing has been used for the clinical diagnosis of cancer. For example, the FoundationOne CDx panel can detect alterations in 324 cancer-related genes. Similarly, the OncoGuide NCC oncopanel system detects alteration in 114 genes [25]. For solid tumors, these assays are conducted using FFPE tissue sections (10 slices of 4–5 µm thickness, 16–25 mm^2^ area per slice, and >20% tumor content). Therefore, improved DNA extraction methods from FFPE tissue sections would increase the sensitivity of these analyses and reduce the minimal specimen requirements.

Besides clinical usage, targeted gene panel sequencing is useful in basic biomedical research. It enables cost-effective analysis of hundreds of samples to maximize their statistical power. Archival FFPE tissue blocks enable efficient sample collection, especially for rare diseases. However, owing to lower quality and quantity of DNA extracted from archival samples poses challenges in molecular genetic analysis. Since we observed that the use of HiTE increases the yield of sequencing library prepared from FFPE tissue sections (Figs 3 and 4), we speculated that the FFPE-DNA extracted using this method would be useful for targeted gene panel sequencing. Therefore, we investigated the feasibility of using HiTE-extracted FFPE-DNA for targeted gene panel sequencing.

**Figure 5:**
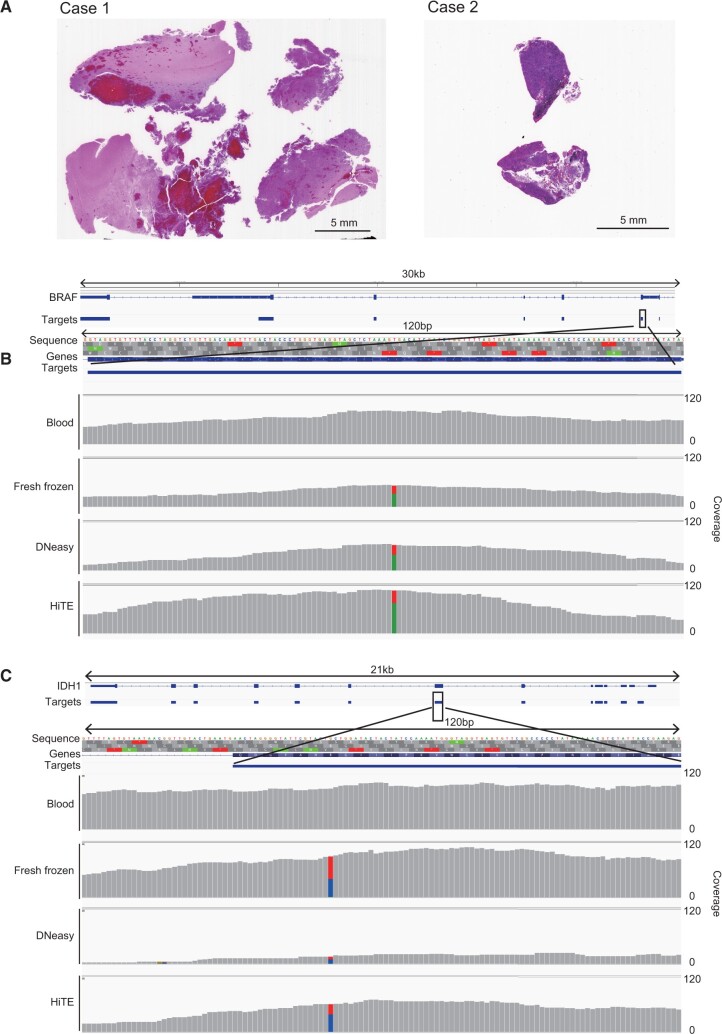
(**A**) Targeted panel sequencing of two glioblastoma cases. Tissues used for the study. The details are provided in Table 1. (**B** and **C**) Representative genome browser shot for *BRAF* (B) and *IDH* (C) genes.

We chose two glioblastoma cases with known mutations in *BRAF* (V600E) and *IDH1* (R132H), respectively (Table 1). These specimens had been stored for 4.4 and 5.7 years, respectively (Table 1). They were selected because in addition to the available FFPE blocks, DNA extracted from fresh frozen tumor tissues and normal peripheral blood was also available from the same patients (Table 1). We prepared FFPE-DNA from a 10-µm tissue slice using DNeasy and HiTE (Fig. 5A and Table 1) and used 30 ng of it for preparing sequencing libraries. Finally, target enrichment was conducted using the xGen Pan-Cancer Hybridization Panel covering 127 cancer-related genes and the obtained target sequences were determined. HiTE yielded three times more DNA than DNeasy (Table 1) and the yield of sequencing library from 30 ng of FFPE-DNA extracted with HiTE was two to three times more than that from DNA extracted with DNeasy (Table 1), corroborating with the results of library preparation described in the previous section (Figs 3 and 4).

While library preparation from FFPE-DNA extracted with HiTE was more efficient than that prepared from DNA extracted with DNeasy, the yield of sequencing library per unit DNA showed a log decrease than that of the library prepared using DNA extracted from fresh frozen tissues (Table 1). This indicates that the sequencing library prepared from FFPE-DNA contains less diversified molecules. Sequencing of a low complexity library produces numerous duplicated reads. After removing the duplicated reads, only shallow read coverage (5.5–6.2× and 14.1–19.1× for DNeasy and HiTE, respectively) was obtained for these FFPE-DNA datasets (Table 1 and Supplementary Fig. S5), thereby making it difficult to identify the known mutations.

Since there was a strong relationship between the deduplicated target read coverage and initial library diversity (Table 1), we surmised that more read coverage would become available by adding more diversified library molecules for target enrichment. Therefore, we next increased the input FFPE-DNA to enhance the library diversity and performed target enrichment again. Notably, because of low FFPE-DNA yield, we could not prepare comparable amounts of libraries with DNeasy samples (Table 1). As a result, using the increased initial DNA input, we could obtain enough read coverage (20.5–29.3× and 77.4–119× for DNeasy and HiTE, respectively) to identify the known mutations in the libraries prepared from FFPE-DNA (Table 1, Fig. 5B and C, and Supplementary Fig. S5). These results strongly indicate the importance of library diversity before PCR amplification for targeted gene panel sequencing. In this regard, HiTE completely surpasses DNeasy because it produced a log higher yield of library per FFPE tissue section than that obtained from the DNeasy procedure (Figs 3 and 4 and Table 1). Based on these results, HiTE would be useful in more sensitive targeted gene panel sequencing.

## Discussion

In the present study, we first investigated the efficiencies of commercially available kits for DNA extraction from FFPE tissue sections. The selected kits gave low yields and poor integrity of DNA, underscoring a huge scope for improvement. Considering the beneficial implications of protracted incubation at high temperatures [16, 17] and formalin scavengers [17], we investigated these parameters to find the optimal condition for extracting DNA from FFPE tissue sections. We established a DNA extraction method called HiTE and improved the DNA recovery from FFPE sections. Besides the yield, the integrity and quality of FFPE-DNA were greatly improved. We applied HiTE for whole-genome and targeted gene panel sequencing of retinoblastoma and glioblastoma, respectively, and showed that HiTE produced superior quality data in both experiments.

### An unexpected effect of Tris in DNA protection

We determined optimal conditions for reverse-crosslinking of FFPE tissue sections. Several studies indicated that incubating the FFPE-tissues at high temperatures improves the yields of FFPE-DNA [11]. However, as indicated by previous studies [4, 13], incubation at temperatures 90°C or higher caused severe DNA damage even with a short incubation period of 1 h (Fig. 2A–C). Therefore, we chose 80°C to achieve a balance between efficient reverse-crosslinking and stability of DNA. Interestingly, while DNA degradation was still observed at 80°C, the coexistence of Tris in the reaction showed a protective effect against DNA degradation in a concentration-dependent manner (Fig. 2D–E).

The use of Tris at high concentrations was based on the known characteristic of the reagent for formalin scavenging effect [17], to enhance the reverse-crosslinking. On the other hand, the DNA protective effect of the Tris was unexpected. There are at least two possible explanations for the mechanism of the phenomenon. The first is that a reduced concentration of free formaldehyde with the scavenger mitigated the DNA damaging effect of the formalin. The other is that Tris has an intrinsic DNA protective effect. Since incubation of DNA in Tris without formalin fixation showed a similar DNA protective effect in a concentration-dependent manner (not shown), the latter possibility is strongly supported. Further investigations are required for a deeper understanding of Tris’s role in providing a DNA protective effect, which may ultimately contribute to the development of a more efficient method for the extraction of FFPE-DNA.

### HiTE would enable more effective utilization of archival tissues

FFPE tissue blocks present a tremendously rich resource for biomedical research. The ease of obtaining the materials required for FFPE blocks makes it easier to maintain their collections, even for rare diseases, in hospitals and research institutions. However, the lack of appropriate techniques and procedures to recover FFPE-DNA undermines the importance of these precious resources in downstream usage for molecular biological diagnostic and research purposes. Using FFPE-DNA from the tissue blocks of 1 month to 20 years of storage, we showed that HiTE can yield better and bigger sequencing libraries than those prepared using DNeasy (Fig. 3D–F). To our surprise, sequencing libraries prepared from old FFPE blocks of 10–20 years of storage produced library with adequate diversity (Fig. 3D and Supplementary Table S2). Although FFPE-DNAs extracted from longer stored blocks were damaged and heavily degraded, our study shows promising and encouraging results for using these blocks for sequencing analysis (Fig. 3B). Moreover, the FFPE-DNA library inserts obtained using HiTE were always larger than those obtained using DNeasy (Fig. 3E). Since the diversity of the sequencing library before PCR amplification is important for detecting known mutations (Fig. 5 and Table 1) and the length of reads is vital for correct mapping of the read to reference genome, the better library yields and larger inserts obtained using HiTE can allow analyzing old FFPE stocks for research and diagnostic purposes.

### Improved yields of and sequencing library with a simplified, cost-effective procedure would be beneficial for many applications

Among the benefits of HiTE is the use of inexpensive reagents and a simplified protocol. The use of Tris buffer, one of the most common reagents used in molecular genetic laboratories, and requirement of only equipment, a block incubator set at 80°C, makes our process feasible in any basic laboratory setting. Moreover, any laboratory performing next-generation sequencing experiments can easily introduce HiTE in their workflow. Although we used DNeasy columns for DNA purification after reverse-crosslinking, silica column purification is not always necessary. We were also able to successfully recover reverse-crosslinked DNA with alcohol precipitation and solid-phase reversible immobilization [26] (data not shown). Thus, both high-throughput and cost-effective DNA purification from the FFPE tissue sections can be achieved with HiTE.

The sequencing library yield per FFPE tissue section showed a log high increase using HiTE. This high efficiency of preparing sequencing libraries from FFPE sections has the potential to change the genetic and epigenetic analyses of cells with specific phenotypes. Currently, several methods for isolating cells in the field of microscopic view are available, such as laser capture microdissection [27], laser cutting microdissection [28], micromanipulation, and tissue micro-dissection punching system [29] are used for this purpose. However, selecting cells of interest using these technologies would inevitably reduce the number of cells to be analyzed. The genetic and epigenetic analysis of these limited numbers of cells would require an efficient technique for FFPE-DNA extraction. With HiTE, such analyses can become feasibly achievable.

Many applications in molecular biology depend on formaldehyde crosslinking. For example, chromatin immunoprecipitation followed by sequencing (ChIP-Seq) is one of the most used experimental procedures for the analysis of epigenomes [30–32]. In ChIP-Seq, formaldehyde crosslinking of nuclei is the first step, and after target epigenome enrichment with immunoprecipitation, DNA is recovered by reverse-crosslinking. HiTE can improve this reverse-crosslinking step and thus possess a potential of becoming a valuable tool in ChIP-Seq. HiTE is thus expected to provide efficient DNA extraction not only from FFPE sections but also in every application that depends on the formaldehyde crosslinking.

### Further developments required

Despite the successful demonstration of the applicability of HiTE and its potential, several issues still remain to be addressed. The first one is the existence of genomic loci reflective of sequencing. When comparing the sequenced read coverage between DNeasy and HiTE, we observed a more even coverage with HiTE. However, at the megabase scale, the read coverage showed fluctuations with HiTE (Fig. 4A and D and Supplementary Fig. S4). The regions with relatively low read coverage correlated well with those enriched with heterochromatin markers (Supplementary Fig. S4). Therefore, it is speculated that the HiTE protocol described herein is inefficient in dissolving such packed chromatin regions. Further improvements for more efficient recovery of such regions are anticipated in the future.

Another issue is the low yields of sequencing library prepared from FFPE-DNA. Although the library yield from FFPE-DNA prepared with HiTE was a log higher than that from DNA extracted with DNeasy, it was an order lower than the yield obtained using DNA extracted from fresh frozen tissues, indicating the existence of some still unresolved problems (Table 1). Incomplete reverse-crosslinking, DNA lesions induced during the formalin crosslinking and high-temperature incubation, DNA strand breaks induced during the long storage period can be the possible causes. A more efficient reverse-crosslinking, bypassing and removal of lesions, and a strand breakage-tolerant library preparation method would effectively solve these problems, respectively. Further studies are required for the establishment of techniques to address these issues.

## Supplementary data

Supplementary data is available at *Biology Methods and Protocols* online.

## Supplementary Material

bpac014_Supplementary_Data

## Data Availability

Sequence data used in the present study were deposited in the Japanese Genotype-Phenotype Archive (JGA) under the accession number JGAS000520. The budding yeast genomic sequence data were deposited to NCBI SRA with accession numbers SRR18473919 and SRR18473920.
