# Correction to: An efficient procedure for the recovery of DNA from formalin-fixed paraffin-embedded tissue sections

**DOI:** 10.1093/biomethods/bpad003

**Published:** 2023-03-13

This is a correction to: Utako Oba, Kenichi Kohashi, Yuhei Sangatsuda, Yoshinao Oda, Koh-Hei Sonoda, Shouichi Ohga, Koji Yoshimoto, Yasuhito Arai, Shinichi Yachida, Tatsuhiro Shibata, Takashi Ito, Fumihito Miura, An efficient procedure for the recovery of DNA from formalin-fixed paraffin-embedded tissue sections, *Biology Methods and Protocols*, Volume 7, Issue 1, 2022, bpac014, https://doi.org/10.1093/biomethods/bpac014

In the originally published version of this manuscript, the final label on the x-axis in Figure 4A was incorrectly given as 8.0E+8, instead of 8.0E+5.

In Figure 4D, the labels on the x-axis were incorrectly given as 0.0E+0, 5.0E+8, 10.0E+8, 15.0E+8, instead of 0.0E+0, 0.5E+8, 1.0E+8, and 1.5E+8

These errors have been corrected.

