# Supplementary material for: An efficient procedure for the recovery of DNA from formalin-fixed paraffin-embedded tissue sections
Source: Biol Methods Protoc. 2022 Jul 26;7(1):bpac014. doi: 10.1093/biomethods/bpac014 (PMC9351614; doi:10.1093/biomethods/bpac014)
Supplement: bpac014_Supplementary_Data [file bpac014_supplementary_data.pdf]

Supplementary material

An efficient procedure for the recovery of DNA from formalin-fixed paraffin-embedded tissue sections

Utako Oba[1,2], Kenichi Kohashi[3], Yuhei Sangatsuda[4], Yoshinao Oda[3], Koh-Hei Sonoda[4], Shouichi Ohga[2], Koji Yoshimoto[5], Yasuhito Arai[6], Shinichi Yachida[7,8], Tatsuhiro Shibata[6,9], Takashi Ito[1] and Fumihito Miura[1,*]

[1] Department of Biochemistry, Kyushu University Graduate School of Medical Sciences, 3-1-1 Maidashi, Higashi-Ku, Fukuoka 812-8582, Japan

[2] Department of Pediatrics, Kyushu University Graduate School of Medical Sciences, 3-1-1 Maidashi, Higashi-Ku, Fukuoka 812-8582, Japan

[3] Department of Anatomic Pathology, Kyushu University Graduate School of Medical Sciences, 3-1-1 Maidashi, Higashi-Ku, Fukuoka 812-8582, Japan

[4] Department of Ophthalmology, Kyushu University Graduate School of Medical Sciences, 3-1-1 Maidashi, Higashi-Ku, Fukuoka 812-8582, Japan

[5] Department of Neurosurgery, Kyushu University Graduate School of Medical Sciences, 3-1-1 Maidashi, Higashi-Ku, Fukuoka 812-8582, Japan

[6] Division of Cancer Genomics, National Cancer Center Research Institute, 5-1-1 Tsukiji, Chuo-ku, Tokyo, 104-0045, Japan

[7] Department of Cancer Genome Informatics, Graduate School of Medicine, Osaka University, 2-2 Yamadaoka, Suita, Osaka 565-0871, Japan

[8] Division of Genomic Medicine, National Cancer Center Research Institute, 5-1-1 Tsukiji, Chuo-ku, Tokyo 104-0045, Japan

[9] Laboratory of Molecular Medicine, Human Genome Center, The Institute of Medical Science, The University of Tokyo, 4-6-1, Shirokanedai, Minato-ku, Tokyo, 108-0071, Japan

* To whom correspondence should be addressed. Tel: +81-92-642-6100; Fax: +81-92-642-6203; Email: fumihito@med.kyushu-u.ac.jp.

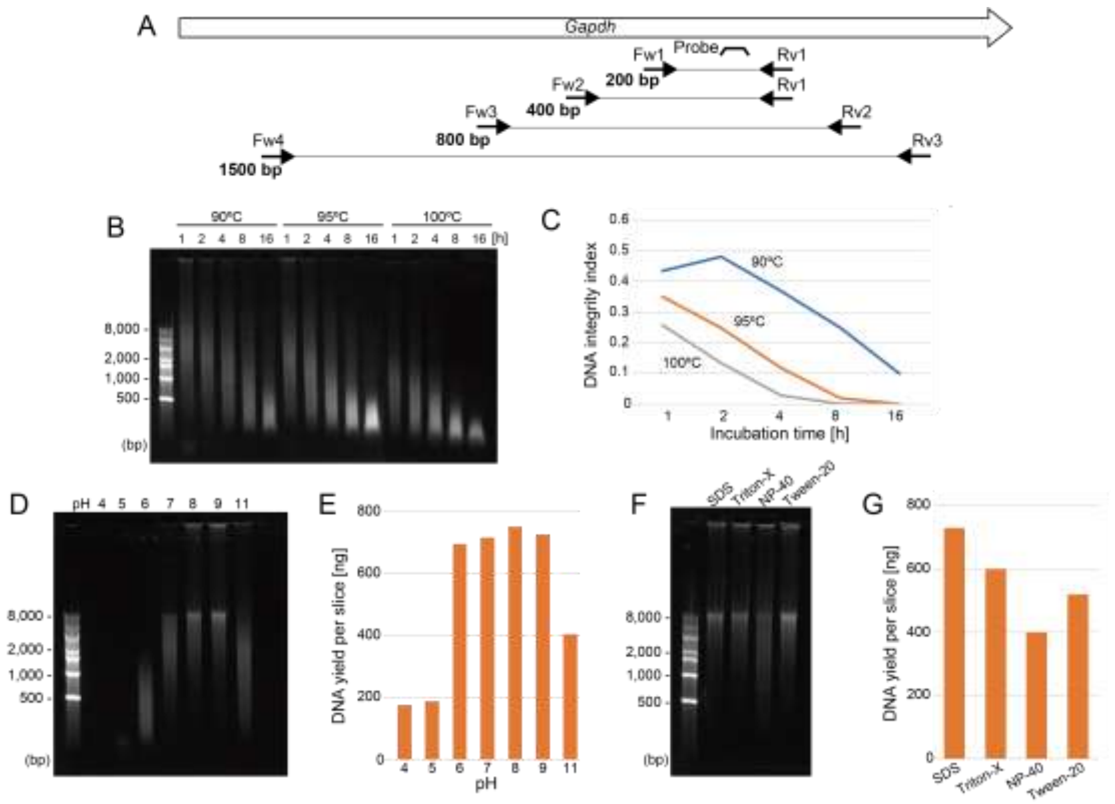

Supplementary Figure S1. Optimization of DNA extraction from formalin-fixed paraffin-embedded (FFPE) tissue sections. A. Position of primers designed on the mouse GAPDH gene and the amplicons are shown. The oligonucleotide sequences are provided in Supplementary Table S1. **B–G.** Effect of incubation temperature and duration (B and C), pH (D and E), and detergents (F and G) on the integrity of DNA extracted from FFPE tissue sections are shown. Representative images showing agarose gel electrophoresis (B, D, and F), DNA integrity index (C), and the yields of the extracted DNA (E and G).

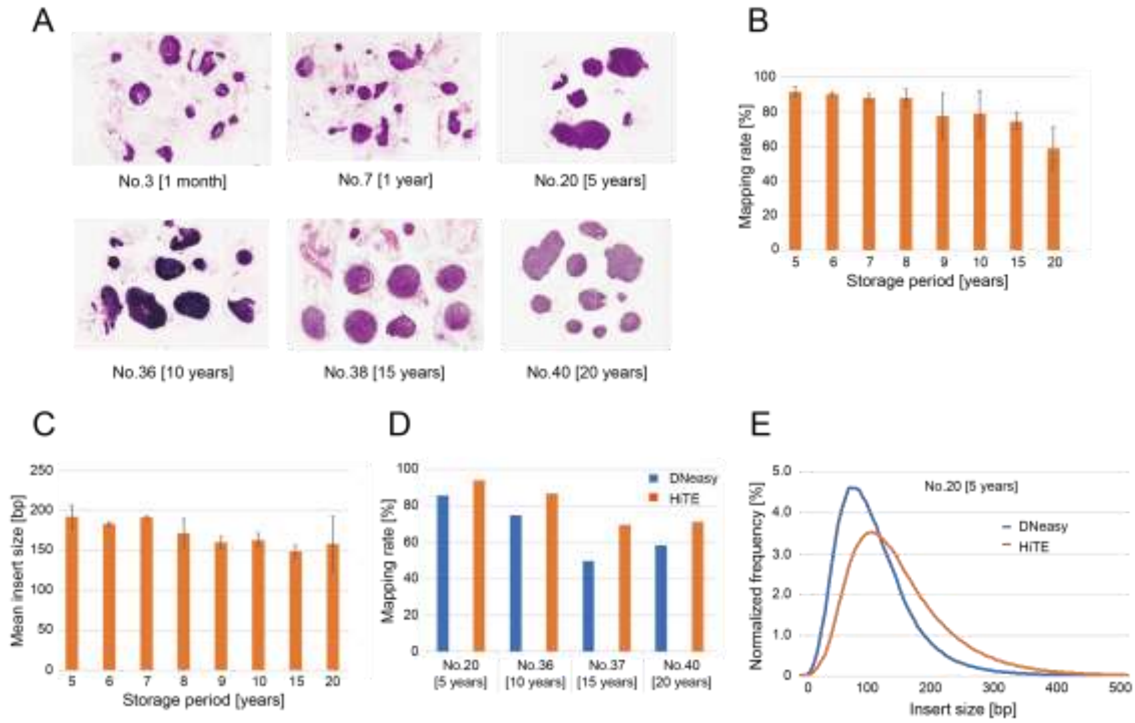

Supplementary Figure S2. DNA extraction from FFPE tissue sections subjected to storage for different periods. A. Representative images of hematoxylin and eosin-stained human normal lymph node used for DNA extraction. **B and C**. Mean mapping rates (B) and insert lengths (C) of reads for HiTE-extracted FFPE-DNA for different storage periods are shown. **D and E.** Comparisons of mapping rates (D) and insert size distributions (E) for sequencing libraries prepared from FFPE-DNA extracted using DNeasy and HiTE. Sequencing libraries were prepared using the ThruPlex DNA-Seq kit.

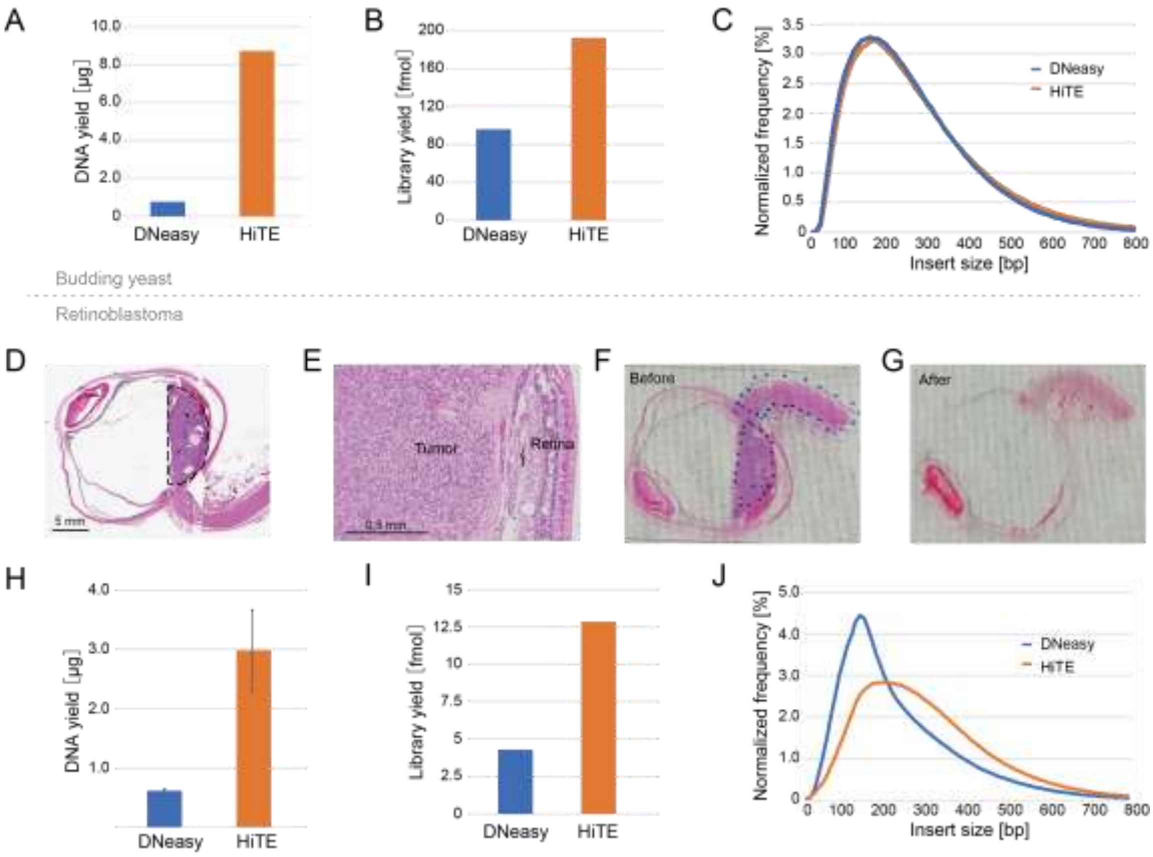

Supplementary Figure S3. HiTE outperforms DNeasy with regard to the FFPE-DNA yield and library preparation. DNA yield (A and H), library yield from unit DNA (B and I), and insert size distribution (C and J) were compared between DNeasy and HiTE. DNA extraction was performed using fixed budding yeast nuclei (A–C) and retinoblastoma tissue (D–J). **D.** The representative images of the hematoxylin and eosin-stained retinoblastoma tissue section used for DNA extraction. **E.** An enlarged view of the tissue. **F and G**. Only tumor tissues were scratched and used for DNA extraction. Representative image before (F) and after (G) scratching the tissues. Sequencing libraries were prepared using the ThruPlex DNA-Seq kit.

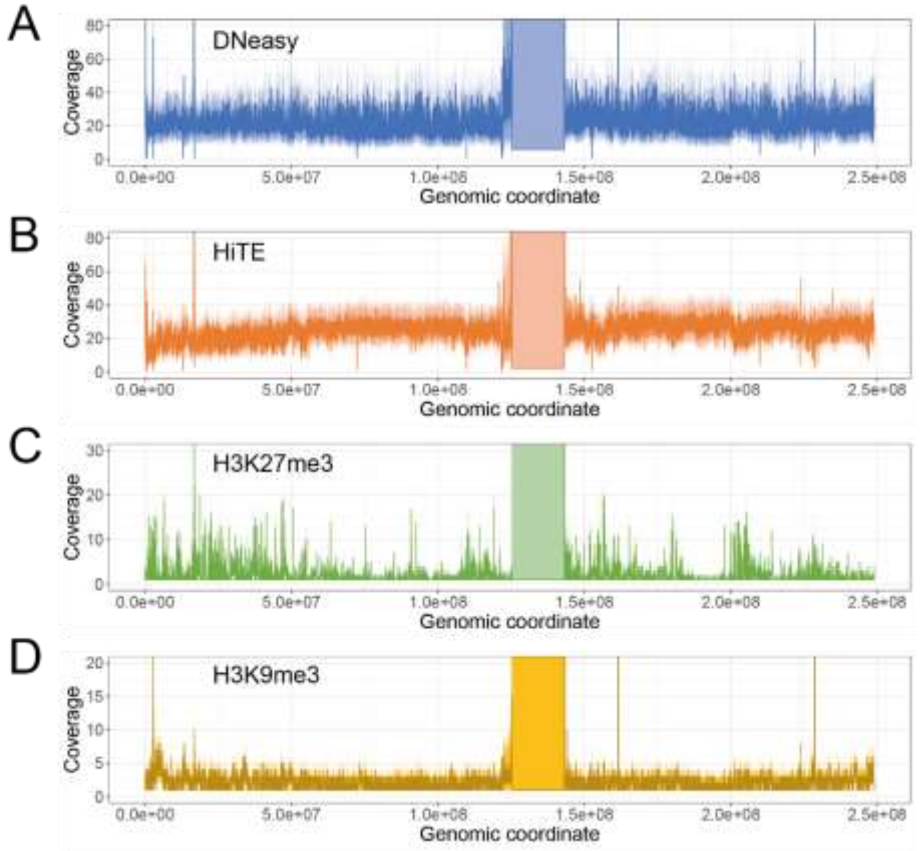

Supplementary Figure S4. Correlated of megabase-sized fluctuation of mapped read coverage of HiTE-extracted FFPE-DNA with heterochromatin markers. A and B. Mapped read coverage of FFPE-extracted using DNeasy (A) and HiTE (B). **C and D.** Mapped read coverages of ChIP-Seq data for two heterochromatin markers H3K27me3 (C) and H3K9me3 (D) (Wong KM et al., GEO accession number: GSE 173125).

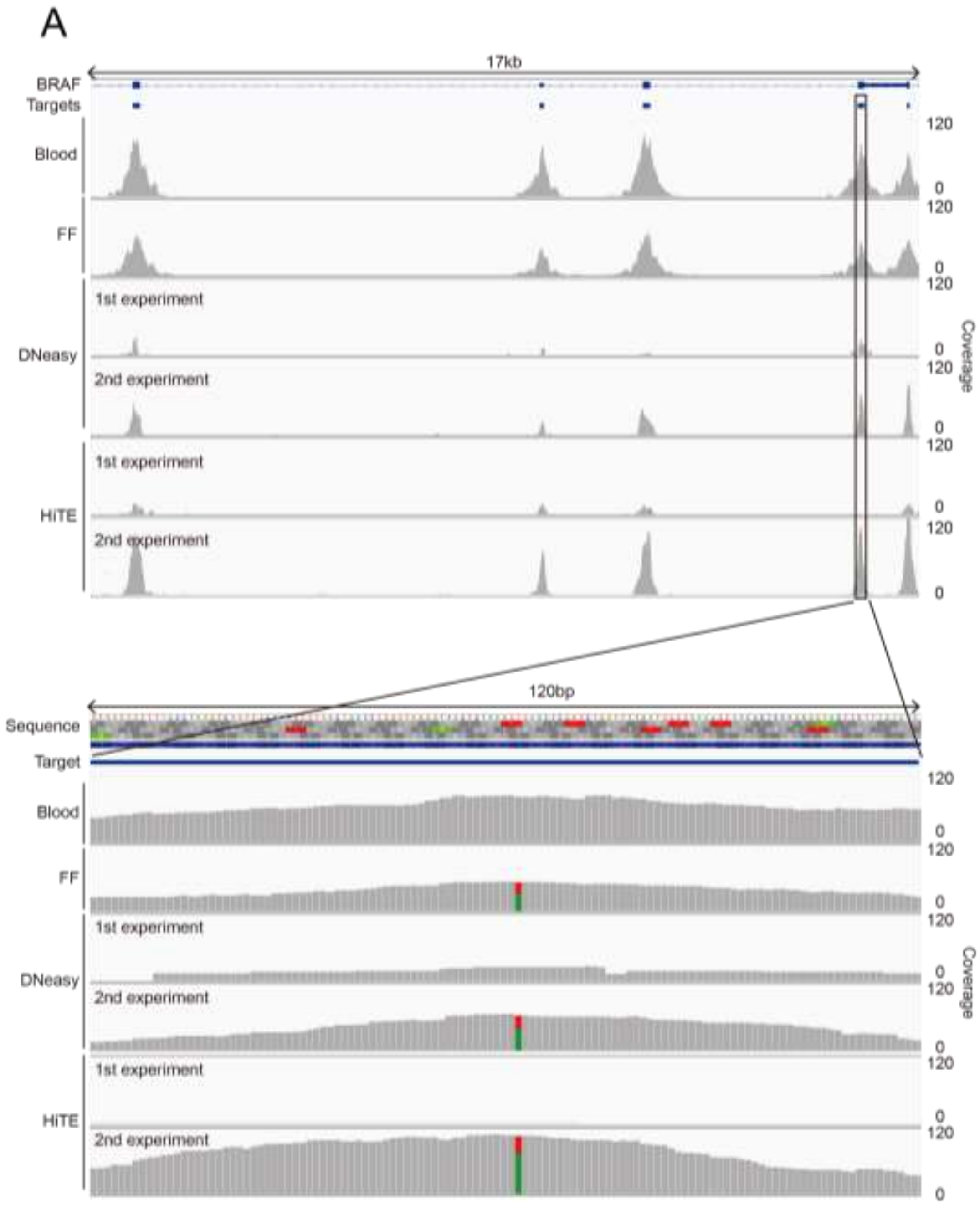

(Continued on the next page)

B

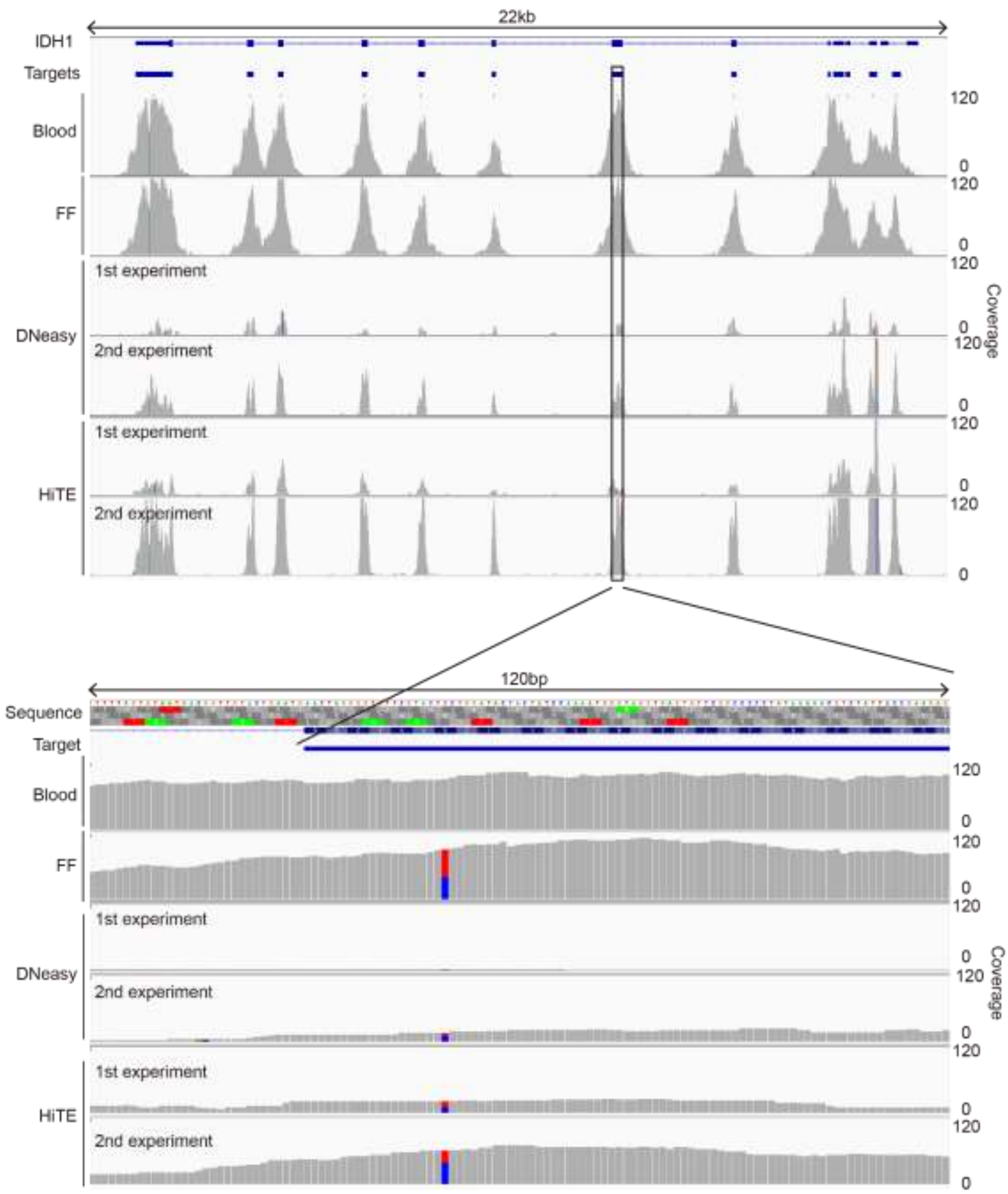

Supplementary Figure S5 Targeted gene panel sequencing of glioblastoma cases with known mutations. When the library diversity was limited (1st experiment), low read coverage after deduplication resulted in the inability to detect the known mutations. On the contrary, the known mutations were detected upon addition of more input DNA (2nd experiment). For details of library yields and mapped read coverages, see Table 1. Cases with a mutation on *BRAF* V600E (A) and *IDH1* R132H (B) are shown.

Supplementary Table S1 Oligonucleotides used for quantitative polymerase chain reaction (qPCR)

Target genome	Target gene	Usage	Name	Oligonucleotide sequence and modifications
Mus musculus	*Gapdh*	Primer	Fw1	5′ -GCCAAGTATGATGACATCAAGAAGG-3′
			Fw2	5′ -GAACATCATCCCTGCATCCACTG-3′
			Fw3	5′ -CAAGGTCATCCATGACAACTTTGG-3′
			Fw4	5′ -CAGTGGCAAAGTGGAGATTGTTG-3′
			Rv1	5′ -CCACATACCAGGAAATGAGCTTGAC-3′
			Rv2	5′ -GTTTCTTACTCCTTGGAGGCCATGTAG-3′
			Rv3	5′ -GTGCAGCGAACTTTATTGATGGTATTC-3′
		Probe	Probe 1	5′ -FAM-CCACCTTCGATGCCGGGGCTGGCATTGC-TAMRA-3′
Human	*GAPDH*	Primer	200 bp-Fw	5′ -ACCTGCCAAATATGATGACATCAAG-3′
			200 bp-Rv	5′ -TGAGCTTGACAAAGTGGTCGTTGAG-3′
		Probe	Probe 2	5′ -FAM-CATCCTGGGCTACACTGAGCACCAGGTGGT-TAMRA-3′

Supplementary Table S2 Comparison of DNeasy and HiTE with regard to FFPE-DNA yield, amplifiable DNA fragments, and sequencing library preparation

Sample No.	Storage period	DNA yield per slice (μg)		Relative amount of 200 bp fragment		WGS library from 2 ng DNA (fmol) (sonicated DNA*)	
		DNeasy	HiTE	DNeasy	HiTE	DNeasy	HiTE
1	1 month	4.770	6.875	0.374	0.522	0.10 (3.82)	0.20 (5.52)
2	1 month	1.350	4.670	0.464	0.525	0.20 (5.27)	0.25 (5.31)
3	1 month	8.850	12.300	0.432	0.762	0.05 (5.55)	0.20 (8.58)
4	6 months	1.880	9.250	0.251	0.412	0.20 (3.22)	0.45 (5.67)
5	6 months	0.640	3.680	0.287	0.415	0.20 (2.04)	0.45 (5.73)
6	6 months	2.790	8.700	0.205	0.405	0.40 (1.43)	0.65 (4.51)
7	1 year	1.490	11.900	0.250	0.299	0.40 (2.33)	1.00 (4.00)
8	1 year	11.100	22.900	0.261	0.334	0.25 (2.33)	0.45 (4.31)
9	1 year	3.540	10.300	0.223	0.315	0.25 (2.16)	0.50 (3.45)
10	2 years	0.580	2.460	0.067	0.109	0.75	1.30
11	2 years	1.360	4.270	0.096	0.204	0.60	0.70
12	2 years	2.220	4.780	0.028	0.094	0.70	2.90
13	3 years	0.281	0.890	0.004	0.009	0.70	2.30
14	3 years	7.350	17.900	0.089	0.143	0.55	1.25
15	3 years	0.755	2.190	0.036	0.095	1.50	2.50
16	4 years	0.965	1.470	0.007	0.027	1.65	4.40
17	4 years	0.266	0.910	0.008	0.048	0.75	2.05
18	4 years	5.100	10.200	0.039	0.084	1.40	2.80
19	5 years	1.270	3.460	0.045	0.067	1.70	2.60
20	5 years	2.190	6.200	0.032	0.072	0.70	1.35
21	5 years	0.446	1.950	0.006	0.019	1.05	2.05
22	6 years	0.297	1.130	0.008	0.020	0.35	0.85
23	6 years	0.338	2.880	0.013	0.023	2.40	5.40
24	6 years	0.328	6.600	0.007	0.022	1.20	1.85
25	7 years	0.115	0.760	0.005	0.023	0.30	2.00
26	7 years	0.144	1.420	0.013	0.024	1.50	1.65
27	7 years	0.103	0.760	0.017	0.041	1.55	2.05
28	8 years	1.580	2.130	0.011	0.031	2.60	5.65
29	8 years	4.100	5.200	0.002	0.007	2.00	4.45
30	8 years	3.620	6.550	0.065	0.080	2.85	5.25
31	9 years	1.615	2.100	0.006	0.013	2.50	6.30
32	9 years	4.670	5.050	0.001	0.008	1.60	3.40
33	9 years	5.700	4.430	0.001	0.001	0.70	1.85
34	10 years	1.560	3.450	0.003	0.010	0.95	2.40
35	10 years	1.460	2.480	0.002	0.001	0.65	1.75
36	10 years	2.780	3.680	0.002	0.007	1.20	3.00
37	15 years	0.414	0.635	0.001	0.001	0.55	1.45
38	15 years	1.490	4.540	0.001	0.002	0.70	1.25
39	15 years	1.270	2.870	0.013	0.010	1.20	2.70
40	20 years	0.580	0.384	0.001	0.004	0.55	1.40
41	20 years	0.164	0.132	0.002	0.001	0.25	0.45
42	20 years	0.498	0.525	0.000	0.000	0.35	1.10

* The yields of libraries prepared from sonicated FFPE-DNA.
